# Decoding age-stratified mutational landscapes in CNS lymphoma via genomic and survival profiling for precision oncology

**DOI:** 10.1016/j.gendis.2025.101934

**Published:** 2025-11-17

**Authors:** Jiawen Chen, Hongwei Liu, Qisheng Tang, Huihui Chai, Peng Wang, Ruize Zhu, Tong Chen, Tianwen Li, Zhifeng Shi

**Affiliations:** a Department of Neurosurgery, Huashan Hospital, Fudan University, Shanghai, 200040, China; b Department of Neurosurgery, Xiangya Hospital, Central South University, Changsha, Hunan, 410008, China; c Hunan International Scientific and Technological Cooperation Base of Brain Tumor Research, Xiangya Hospital, Central South University, Changsha, Hunan, 410008, China; d The National Key Clinic Specialty, The Engineering Technology Research Center of Education Ministry of China, Guangdong Provincial Key Laboratory on Brain Function Repair and Regeneration, Department of Neurosurgery, Zhujiang Hospital, Southern Medical University, Guangzhou, Guangdong, 510282, China; e Department of Hematology, Huashan Hospital, Fudan University, Shanghai, 200040, China

Primary central nervous system lymphoma (PCNSL), a rare extranodal non-Hodgkin lymphoma, is most commonly a diffuse large B-cell lymphoma (DLBCL). Although treated with surgery, radiotherapy, and chemotherapy, its high recurrence rate leads to an unfavorable prognosis.^1^ Recent advances in high-throughput molecular profiling, particularly whole-exome sequencing (WES) and genome-wide association studies (GWASs), have significantly transformed our understanding of the molecular pathogenesis of PCNSL. Recurrent somatic alterations in genes such as *IGLL5*, *PIM1*, *MYD88*, *BTG2*, *PCLO*, *KMT2D*, and *BTG1* have been implicated in PCNSL oncogenesis.^1^^,^^2^ However, existing WES and GWAS in PCNSL have predominantly focused on older populations, leaving young patients, particularly those under 40 years of age, markedly under-represented. This data gap has created a critical barrier to understanding the full spectrum of molecular heterogeneity across age groups.

This study reviewed the records of 1420 pathologically confirmed PCNSL patients treated at our hospital from 2015 to 2025. The ages of these patients ranged from 7 to 88 years, with a median age of 56.5 years. Although there is no universally accepted age range for defining young adults in neuro-oncology, we adopted a cutoff of 40 years based on precedents from previous studies on brain tumors, including PCNSL and glioma.^1^^,^^3^^,^^4^ Accordingly, only 69 patients (4.85%) were < 40 years old (Fig. S1A and Table S1). Of the 69 young patients included in this cohort, comprehensive laboratory analyses, WES, and prospective survival follow-up assessments were successfully completed in 19 rigorously selected cases during the study period. A geriatric control cohort (*n* = 18) was randomly selected from the eligible population as a control. Based on these findings, this retrospective study included 37 consecutive patients with histopathologically confirmed PCNSL who underwent WES. All patients had confirmed diagnoses of PCNSL and no evidence of Epstein–Barr virus (EBV) infection (Fig. S1B). Follow-up was conducted until January 2025, and sequencing was successfully completed for all formalin-fixed paraffin-embedded tumor specimens. Baseline demographic and clinical data—including age, sex, cell of origin, Ki-67 index, Karnofsky Performance Status (KPS) score, Eastern Cooperative Oncology Group (ECOG) performance status, Memorial Sloan-Kettering Cancer Center (MSKCC) score, treatment modality, tumor location, and extent of resection—were retrospectively collected from medical records and are summarized in Table S2. Written informed consent was obtained from all participants, and the study was approved by the Ethics Committee of Huashan Hospital of Fudan University. In this study, all 9 disease-related fatalities occurred in patients aged ≥ 40 years. Univariate and multivariate analyses showed that overall survival (OS) in patients with PCNSL was not significantly associated with sex, extent of tumor resection, Ki-67 proliferation index, KPS score, ECOG performance status, or radiotherapy. Furthermore, adjuvant chemotherapy was associated with a significant improvement in OS, consistent with the existing literature (Fig. 1A). Notably, patients under 40 years of age demonstrated significantly longer OS than older patients (Fig. 1B and C).

**Figure 1:**
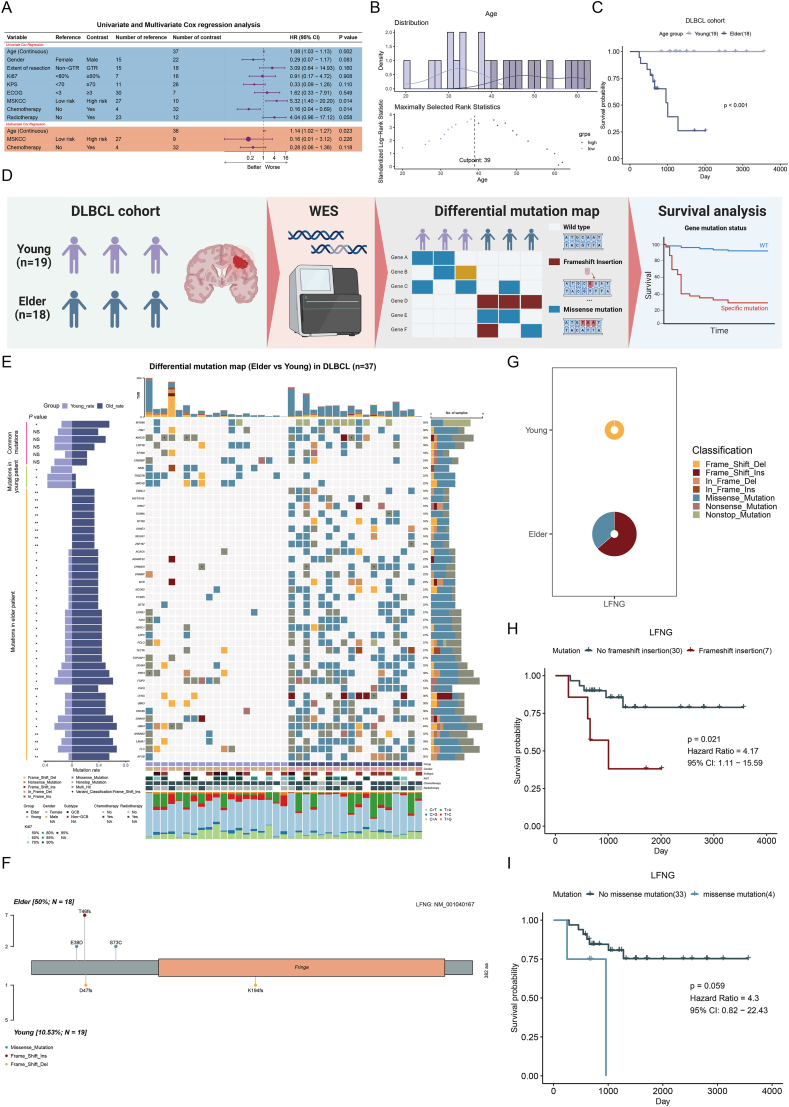
Clinical characteristics and mutation landscapes between young-onset and elder-onset primary central nervous system lymphoma (PCNSL). **(A)** Univariate and multivariate Cox regression analyses identifying age, MSKCC score, and chemotherapy as independent prognostic factors for overall survival. **(B)** Age distribution of patients with PCNSL, stratified into high-risk (≥ 40 years) and low-risk (< 40 years) groups. **(C)** Kaplan–Meier survival curves demonstrating significantly better overall survival in younger patients (< 40 years) than in older patients (≥ 40 years). **(D)** Overview of the study design, cohort characteristics (*n* = 37), and sequencing workflow. **(E)** Most frequently mutated genes in the PCNSL cohort. **(F)** Schematic representation of *LFNG* mutations across the gene domain. **(G)** Pie chart illustrating the distribution of *LFNG* mutation types. The size of the pie representing the number of mutations. **(H)** Kaplan–Meier survival curve showing significantly shorter overall survival in patients with *LFNG* frameshift insertion mutations. **(I)** Kaplan–Meier survival curve showing no significant association between *LFNG* missense mutations and OS.

Based on this observation, the 37 patients were stratified into a younger group (*n* = 19) and an older group (*n* = 18), and WES was performed on their paraffin-embedded tissue samples (Fig. 1D). Owing to sample size constraints, the analysis focused on genes mutated in at least five patients. First, genomic analysis identified several recurrently mutated genes in PCNSL, including *PIM1*, *KMT2D*, *LRP1B*, *EP300* and *CREBBP* that showed comparable mutation frequencies between age groups (Fig. 1E). Strikingly, *MYD88* mutations demonstrated significant age-associated enrichment and were markedly more prevalent in elderly patients than in younger individuals (Fig. 1E). Importantly, survival analysis confirmed that *MYD88* mutation was associated with significantly poorer clinical outcomes (Fig. S2A), corroborating previous reports in the literature^1^ and further reinforcing its value as a predictive biomarker in PCNSL. In addition to the recurrently mutated genes, 40 genes showed significant differences in mutation frequency between the two groups (Fig. 1E). Subgroup analysis showed that the most commonly mutated genes in the younger group were *NEBL*, *THSD7B* and *UNC5D*, whereas in the older group, *FLG*, *UBR4* and *FSIP2* were the most frequently mutated genes (Fig. 1E). These mutations primarily consisted of single-nucleotide variants and insertions/deletions (Fig. S1C). In particular, a significantly lower mutational burden was detected in younger patients (Fig. 1D). Gene ontology enrichment analysis further revealed that the differentially mutated genes were primarily involved in biological processes related to double-stranded DNA repair and the DNA damage response, suggesting that dysregulation of these pathways may contribute to the development and progression of PCNSL (Fig. S1E).

Building on the 40 previously identified differentially mutated genes, further analysis was conducted to explore their association with OS in patients with PCNSL. Survival analysis revealed that mutations in 19 genes (*ACACA*, *ADAMTS2*, *APOB*, *DNMBP*, *EPPK1*, *FAT2*, *FLG*, *HERC1*, *HIST1H1E*, *LAMA1*, *LRP2*, *MYD88*, *MYH7*, *PCLO*, *PCSK5*, *SETX*, *SP100*, *TECTA*, and *ZFHX4)* were significantly associated with patient prognosis. Patients harboring these mutations had significantly shorter OS than those without such mutations (Fig. S2A), suggesting that these genes may contribute to the pathogenesis and progression of PCNSL and may serve as potential prognostic biomarkers. Given the unique immune microenvironment of PCNSL, characterized by infiltrating immune cells (B cells, T cells, macrophages and dendritic cells) and resident stromal components (oligodendrocytes and meningeal cells) that collectively regulate tumor progression,^5^ we systematically investigated the potential immune-related functions of the 40 differentially mutated genes. This analysis identified four genes involved in immune regulation: *MYD88*, *APOB*, *SP100*, and *LFNG* (Fig. S2B). Specifically, *MYD88*, *APOB*, and *SP100* mutations are more frequent in older patients and are associated with significantly shorter OS than those of non-mutated cases (Fig. 1E; Fig. S2A). In contrast, mutations of *LFNG* did not exhibit a statistically significant association with OS in this cohort (Fig. S2C). We further investigated whether specific types of *LFNG* mutations might influence prognosis. In this cohort, age-related differences in *LFNG* mutation patterns were observed. Among younger patients, only two (10.5%) harbored *LFNG* mutations, both of which were frameshift deletions. In contrast, nine patients (50.0%) in the older group had *LFNG* mutations, including missense mutations and frameshift insertions (Fig. 1F and G). Notably, survival analysis demonstrated that the type of *LFNG* mutation significantly impacted prognosis. In particular, patients with frameshift insertion mutations had significantly shorter OS than those without such mutations (Fig. 1H), whereas missense mutations showed no significant association with OS (Fig. 1I). These findings suggest that specific *LFNG* mutation types, particularly frameshift insertions, may serve as prognostic markers in PCNSL.

In summary, this study confirms age as an independent prognostic factor in patients with PCNSL and, for the first time, systematically characterizes the mutational landscape of younger and older PCNSL patients, identifying several gene mutations significantly associated with OS. However, the study has several limitations. First, the relatively small sample size in this cohort may have influenced the results. Second, although several gene mutations, particularly in *LFNG*, were associated with OS, their functional roles and molecular mechanisms have not yet been validated through *in vitro* or *in vivo* experiments.

## CRediT authorship contribution statement

**Jiawen Chen:** Writing – original draft, Conceptualization. **Hongwei Liu:** Writing – original draft. **Qisheng Tang:** Writing – review & editing. **Huihui Chai:** Data curation. **Peng Wang:** Software, Conceptualization. **Ruize Zhu:** Data curation. **Tong Chen:** Data curation. **Tianwen Li:** Conceptualization. **Zhifeng Shi:** Conceptualization.

## Ethics declaration

The study was reviewed and approved by the Ethics Committee of Huashan Hospital of Fudan University (Approval number: 2022-913), and informed consent was obtained from the patients.

## Data availability

The datasets used and/or analyzed during the current study are available from the corresponding author upon reasonable request.

## Funding

This study was supported by the 10.13039/501100012166National Key Research and Development Program of China (No. 2022YFF1202804, 2023YFC2510000, 2024YFA1210201), the 10.13039/501100001809National Natural Science Foundation of China (No. 82373018, 82473295), the Excellent project of 10.13039/100017950Shanghai Municipal Health Commission (China) (No. 20234Z0009), the Medical innovation research project of Shanghai Science and Technology Commission (China) (No. 23Y11906200), the Dawn Program of Shanghai Education Commission (China) (No. 23SG07), the Fudan University Medical Engineering Integration Project (China) (No. IDH2310155), the 10.13039/501100002858China Postdoctoral Science Foundation (No. 2023TQ0072, 2023M740676) and the Non-profit Central Research Institute Fund of Chinese Academy of Medical Sciences (No. 2024-JKCS-06).

## Conflict of interests

None declared. Figures were created by Peng Wang using BioRender software (https://biorender.com).
