# Supplementary material for: Decoding age-stratified mutational landscapes in CNS lymphoma via genomic and survival profiling for precision oncology
Source: Genes Dis. 2025 Nov 17;13(5):101934. doi: 10.1016/j.gendis.2025.101934 (PMC13122674; doi:10.1016/j.gendis.2025.101934)
Supplement: Multimedia component 1 [file mmc1.docx]

**Supplementary Material**

**
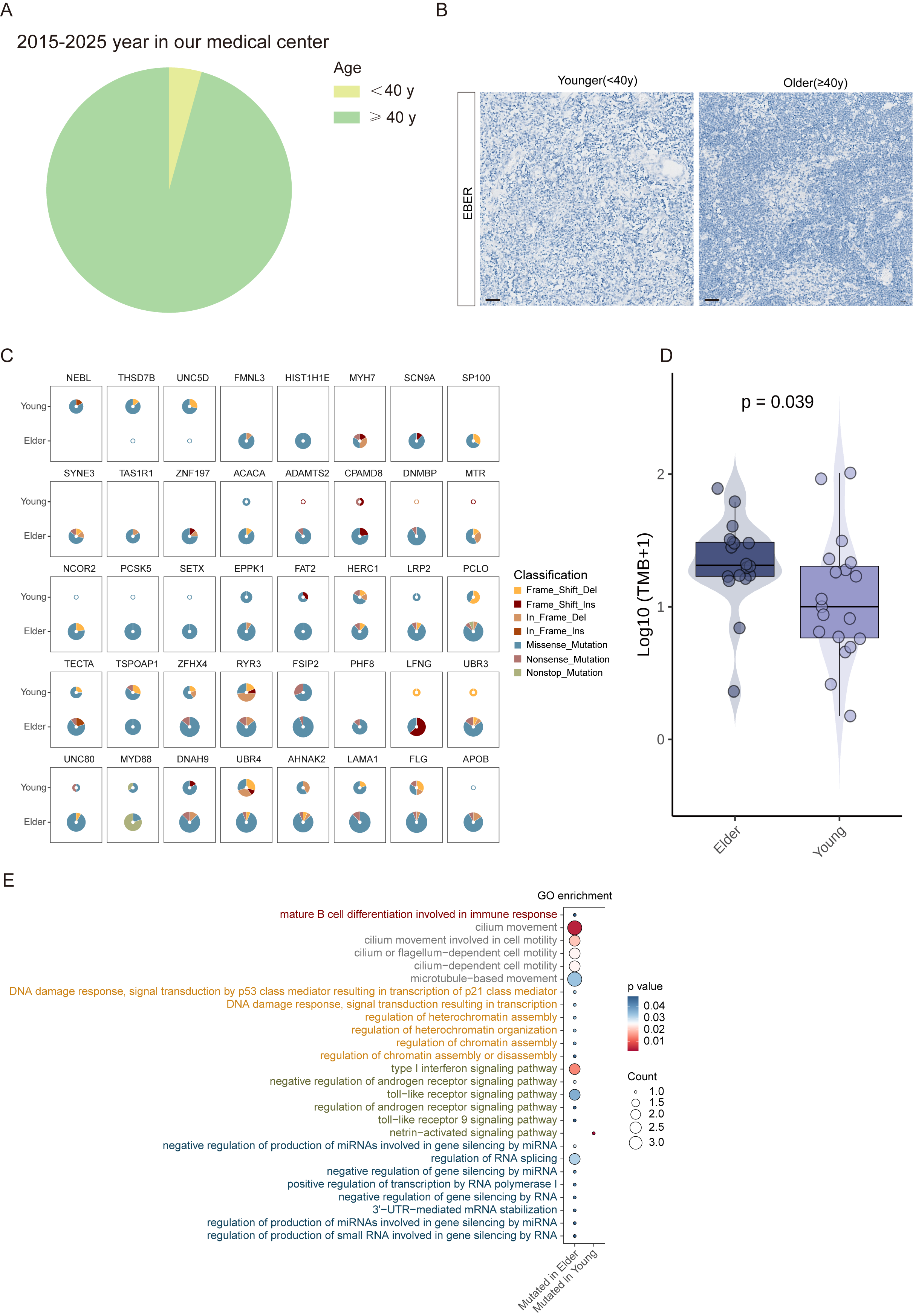
**

**Supplementary Figure ~~S~~1. Age distribution, EBV detection, and mutational profiling in PCNSL.** (A) Pie chart demonstrating the age distribution of central nervous system lymphoma patients treated at our hospital between 2015 and 2025. (B) All enrolled patients demonstrated negative EBV-encoded RNA (EBER) in in situ hybridization analysis. Scale bar = 50 μm. (C) Distribution of mutation types in younger (< 40 years) and older (≥ 40 years) patients. (D) Comparison of mutational burden between younger (< 40 years) and older (≥ 40 years) patients. *P* values, two-sided unpaired Wilcoxon test. (E) GO enrichment analysis of mutated genes in the full cohort.


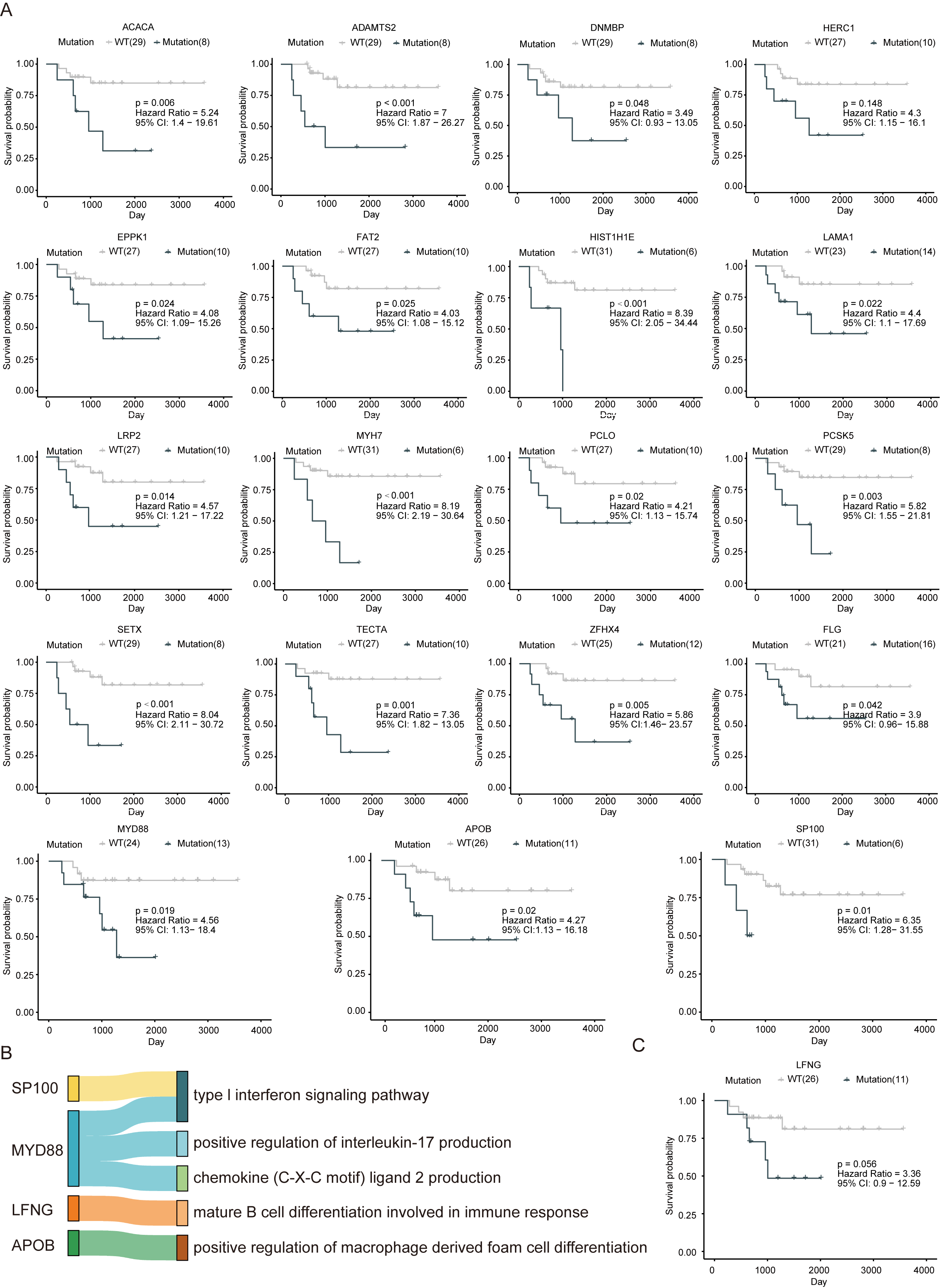


**Supplementary Figure ~~S~~2. Survival impact of gene mutations in PCNSL by age group.** (A) Kaplan-Meier survival analyses for individual mutant genes in younger (< 40 years) and older (≥ 40 years) patients with primary central nervous system lymphoma. (B) Four mutated genes associated with immune microenvironment regulation. (C) Kaplan-Meier survival analyses for *LFNG* in younger (< 40 years) and older (≥ 40 years) patients.

**Materials and Methods**

**Study Population and Clinical Characteristics**

Thirty-seven patients with PCNSL were recruited from Huashan Hospital of Fudan University between March 2015 and May 2025. Inclusion Criteria: (1) Histopathologically confirmed diagnosis of PCNSL; (2) Availability of formalin-fixed paraffin-embedded (FFPE) tumor tissue samples obtained at diagnosis that were suitable for whole-exome sequencing (WES); (3) Complete and traceable clinical medical records; (4) Absence of known congenital or acquired severe immunodeficiency; (5) Written informed consent voluntarily provided by the patient or their legal representative; (6) Negative EBER in situ hybridization results in tumor tissue. Exclusion Criteria: (1) Ambiguous or controversial pathological diagnoses; (2) Poor sample quality, including low tumor cell content, severely degraded DNA, or insufficient material for WES library construction; (3) Loss to follow-up; (4) Positive EBER in situ hybridization results in tumor tissue; (5) Comorbid Medical Conditions: Presence of severe or unstable comorbid conditions that could pose an additional risk or interfere with study assessments (e.g., uncontrolled hypertension, congestive heart failure, severe cardiac arrhythmias, active systemic infections). History of other active malignancies within the past 5 years. History of significant neurological or psychiatric disorders that could impair the ability to provide informed consent or comply with study procedures; (6) Other Interfering Factors: History of chronic alcohol or drug abuse within the past year. Individuals under legal or administrative supervision. All patients had confirmed diagnoses of PCNSL and no evidence of immunodeficiency. Follow-up was conducted until January 2025, and sequencing was successfully completed for all formalin-fixed paraffin-embedded tumor specimens. Baseline demographic and clinical data—including age, sex, cell of origin, Ki-67 index, KPS score, ECOG performance status, MSKCC score, treatment modality, tumor location, and extent of resection—were retrospectively collected from medical records and are summarized in Supplementary Table S2. Written informed consent was obtained from all participants, and the study was approved by the Ethics Committee of Huashan Hospital of Fudan University.

**DNA Isolation**

Genomic DNA was extracted from brain tissue samples of 37 patients with primary central nervous system diffuse large B-cell lymphoma using a formalin-fixed, QIAamp DNA FFPE Tissue Kit (Qiagen, Hilden, Germany). DNA concentration was measured using the Qubit® 3.0 Fluorometer (Thermo Fisher Scientific).

**WES and Data Processing**

Sequencing was performed using a tumor gene panel on the Illumina NovaSeq 6000 System (Illumina Inc., CA, USA) provided by Sinotech Genomics Co., Ltd, Shanghai. Exome-enriched DNA fragments were captured and amplified using the SureSelect Human All Exon V6 (Agilent Technologies Inc.), following the instructions of the manufacturer. Library quality was assessed using the Agilent 4200 TapeStation (Agilent Technologies Inc.) prior to high-throughput sequencing. After quality control using fastp (v0.20.1), clean reads were aligned to the GRCh37 human reference genome using BWA-MEM (v0.7.17) with default parameters.

**Variant Calling and Mutation Analysis**

According to GATK best practices, pre-processing, including marking PCR duplicates and base quality recalibration, was performed using Picard tools (v.1.141) and GATK (v4.1.9.0). Somatic SNV/INDELs were identified using GATK MuTect2 based on a panel of normal derived from the 1,000 Genomes Project, and GATK FilterMutectCalls was used to filter out unreliable somatic mutations. Then, variants were screened based on following conditions: (1) variants were marked as PASS by FilterMutectCalls; (2) variant sites with the sequencing depth in the tumor samples ≥ 30; (3) variants with the mutation allele frequency in the tumor samples ≥ 0.05; (4) variants with the allele depth in the tumor samples ≥ 3; (5) variants with the mutation allele frequency ≤ 0.01 in database of 1,000 genomes project, 1000 genome East Asian or genomAD; (6) variants were exonic mutations. We defined genes mutated in at least five patients and differential mutation frequency between two groups ≥ 0.3 with *P* value < 0.05 as significantly mutated genes.

**Survival Analysis**

Patients were divided into different groups according to a binary categorical variable for OS analysis. For continuous variable age, the maximally selected rank statistics were applied to determine the optimal cut-point related to the outcome by the R package survminer (v0.4.9). The univariate and multivariate Cox proportional hazard models were executed by the R package ezcox (v1.0.2).

**Functional Enrichment Analysis**

Gene Ontology (GO) enrichment analysis based on the differentially mutated genes was performed using the R package clusterProfiler (v4.7.1). Biological processes with a *P* value < 0.05 were considered significant enrichment.

**Statistical Analysis**

All the data analysis and graph generation were completed in R (v4.1.3), Adobe Photoshop software, and BioRender.com. Hazard ratios (HRs) with 95% confidence intervals (CI), log-rank *P* values, and Kaplan–Meier curves were calculated and plotted by the R package survival (v3.3–1) and survminer (v0.4.9). Continuous variables fitting a normal distribution between binary groups were compared using a two-tailed Student's t test, otherwise Wilcoxon test. A *P*-value of <0.05 was considered statistically significant.
